# Temperature as a Neglected Variable in Cancer: Cell-Intrinsic Thermal Sensitivity, Fever-Range Immunity, and the Febrile Paradox

**DOI:** 10.3390/cells15211967

**Published:** 2026-10-28

**Authors:** Michael Linnebacher, Sandra Schwarz, Clemens Schafmayer

**Affiliations:** 1 Molecular Oncology and Immunotherapy, Clinic of General Surgery, University Medical Center Rostock, 18057 Rostock, Germany; sandra.schwarz@med.uni-rostock.de; 2 Clinic of General Surgery, University Medical Center Rostock, 18057 Rostock, Germany

**Keywords:** temperature, fever, fever-range hyperthermia, heat-shock response, proteostasis, tumor microenvironment, antitumor immunity, thermosensitivity, colorectal cancer, cancer models

## Abstract

Temperature is a fundamental physical variable affecting protein folding, proteostasis, cell-cycle kinetics, and immune function, yet cancer cell biology is performed almost exclusively at a fixed 37 °C, and distinct thermal phenomena are often conflated under the single term “hyperthermia.” This review examines the biology of mild, fever-range temperature excursions of approximately 38–41 °C, which are mechanistically distinct from externally induced hyperthermia. We synthesize three strands of evidence that have rarely been considered together. First, tumor cells display heterogeneous thermal sensitivity, with differences in growth, cell-cycle responses, and thermal tolerance that may reflect intrinsic molecular features and proteostatic reserve, including the heat-shock response mediated by HSF1 and HSP70/HSP90. Second, fever-range temperatures remodel antitumor immunity across innate and adaptive compartments, affecting immune-cell trafficking, membrane organization, metabolism, and tumor–immune recognition. Third, the clinical record appears controversial: febrile infections have been associated with lower subsequent cancer risk, whereas postoperative fever has been associated with worse prognosis. We argue that these observations cannot be interpreted without considering host state, timing, and the biological context in which temperature elevation occurs. The three evidence streams are of unequal strength: cell-intrinsic thermal sensitivity and fever-range immune effects rest on direct experimental data, much of it from colorectal cancer, which we adopt as the principal proof-of-concept model, whereas the clinical associations are largely retrospective, and we treat them with correspondingly different confidence. We therefore frame a central testable question: is cell-intrinsic thermal sensitivity a cause or a correlate of fever’s effects on cancer? We propose an experimental agenda that separates cell-autonomous thermal responses from host-mediated effects and establishes temperature as an explicit, biologically informative variable in cancer research.

## 1. Introduction: Temperature as a Neglected Variable in Cancer Biology

Cancer biology has mapped the genetic, metabolic, and immune determinants of malignancy in remarkable detail, yet it largely treats temperature as a fixed background condition. Almost all tumor cell biology is studied at 37 °C, and the field often groups several distinct phenomena under the single term hyperthermia. This is a conspicuous gap, because temperature is not passive. Solid tumors generate heat through vascularization and metabolism, and infrared imaging has long detected many tumors as warmer than adjacent tissue, whereas necrotic cores and some tumor types appear cooler. The tumor is therefore better understood as a heterogeneous thermal landscape than as a body at a single temperature. Infrared thermography has been investigated as a means of visualizing spatial temperature distributions in oncology, although surface imaging does not establish deep intratumoral temperatures [1].

We do not claim that tumors maintain a stably elevated temperature through their own metabolism. Bulk intratumoral temperature is governed largely by perfusion, and reports of large subcellular thermal gradients remain physically disputed [2,3,4]. The relevant biology here is the thermal excursion, the transient elevation imposed by fever, peritumoral inflammation, or deliberate heating, and the observation that tumors, and the cells within them, can respond to such excursions very differently. The question is not whether heat can kill a tumor, which ablative therapies already exploit, but what the everyday thermal fluctuations associated with illness and inflammation do to a growing cancer and to the immune response mounted against it.

The topic is timely. The past three years have produced a surge of mechanistic work on how fever-range temperatures shape T-cell metabolism and antitumor immunity [5,6], alongside renewed clinical evaluation of whole-body hyperthermia [7,8,9], some of it at temperatures at or above the upper end of the fever range and therefore only partly informative about fever-range biology. These advances have accumulated in partly separate literatures spanning tumor-cell thermosensitivity, fever immunobiology, and fever epidemiology that rarely intersect. The idea itself is old: deliberate fever induction with bacterial toxins produced documented tumor regressions more than a century ago, but this approach was displaced by radiotherapy and chemotherapy and never resolved mechanistically. These strands also converge on an unresolved clinical contradiction: a history of febrile infection is associated with lower cancer risk [10,11], yet postoperative fever predicts worse outcomes. In this review, we integrate these strands, treat the apparent contradiction as a hypothesis-generating framework to be reconciled by host state and timing rather than a paradox to be dissolved, and pose a single organizing question: is the intrinsic thermal sensitivity of the tumor cell a cause or a correlate of fever’s effects on cancer?

## 2. Fever Is Not Hyperthermia: Defining the Fever Range

A recurring source of confusion is the assumption that all routes to an elevated tissue temperature are biologically equivalent. Fever is a regulated state in which pyrogenic signaling raises the hypothalamic set point, and the body actively generates and defends a higher core temperature through thermogenesis and behavioral responses. Externally induced hyperthermia raises tissue temperature without changing the set point, so the organism instead works to dissipate the imposed heat and restore homeostasis. The two therefore share an elevated temperature but differ fundamentally in their neuroendocrine and cytokine contexts [12,13,14].

**Figure 1:**
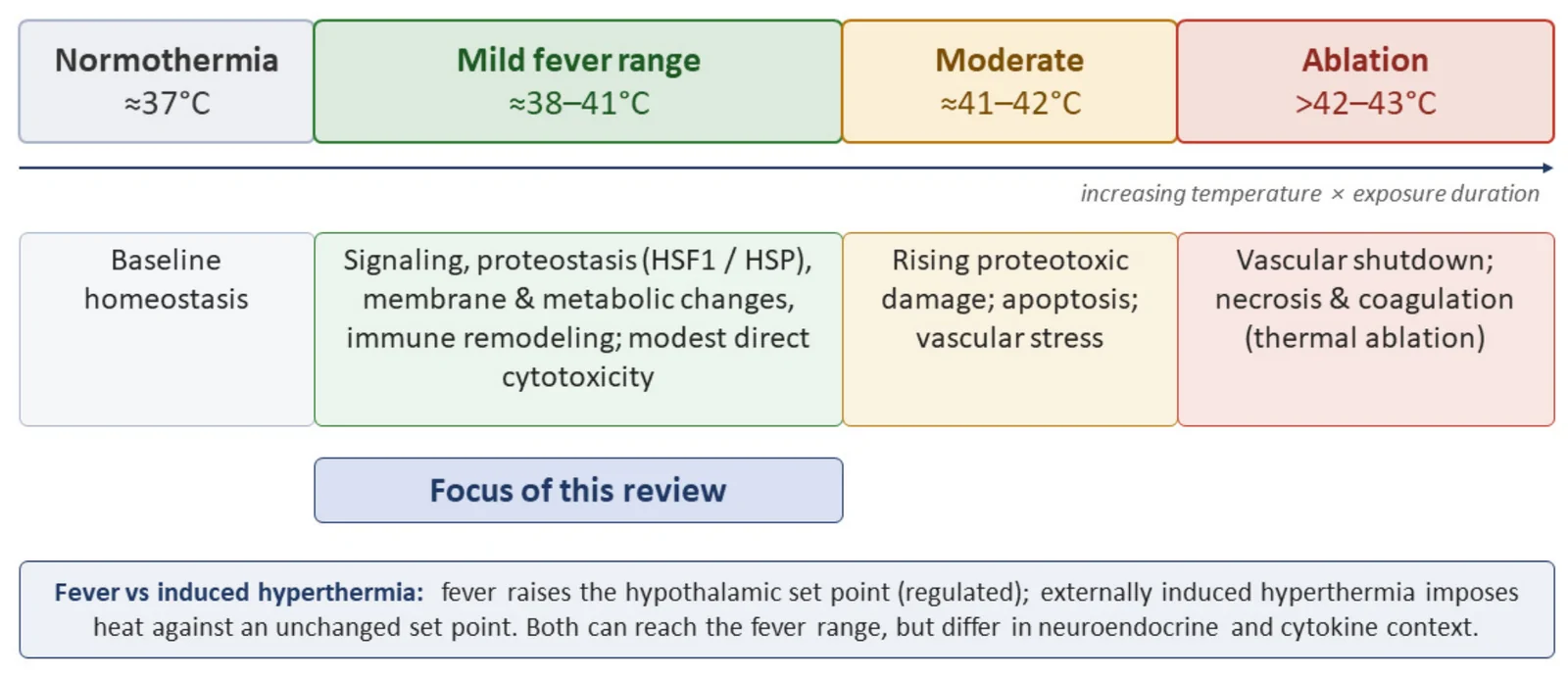
The thermal spectrum in tumor biology. Temperatures spanning normothermia (~37 °C), the mild fever range (~38–41 °C), moderate hyperthermia (~41–42 °C), and ablative heating (>42–43 °C), with the dominant biological effect in each band: largely signaling, proteostatic, metabolic, and immune modulation in the fever range versus progressive cytotoxicity and vascular damage above it, the latter governed by thermal dose rather than peak temperature alone. The distinction between a regulated febrile set-point shift and externally imposed hyperthermia is indicated; this review concerns the mild fever-range band.

It is equally important to distinguish the mild fever range, approximately 38–41 °C, from moderate and ablative heating. At fever-range temperatures, direct cytotoxicity to most tumor cells is limited, whereas effects on cell signaling, proteostasis, metabolism, and immunity become increasingly prominent. Above approximately 41–42 °C, thermal stress becomes progressively more damaging to cells and the tumor vasculature, entering the range exploited by thermal ablation. Critically, these bands are not defined by temperature alone: thermal injury reflects the combination of temperature and exposure duration, conventionally formalized as a thermal dose such as cumulative equivalent minutes at 43 °C, rather than a single fixed temperature threshold [14,15]. Fever-range whole-body hyperthermia can serve as an experimental and clinical surrogate for the elevated temperatures of fever without reproducing the underlying set-point shift, and much of the mechanistic evidence discussed below derives from this approach. This review focuses specifically on the mild fever-range thermal excursion, not on ablative heating (Figure 1).

## 3. Tumor Cells Differ in How They Sense Heat

If tumor cells responded uniformly to mild warming, their intrinsic thermal sensitivity would hold little interest. They do not. When panels of human cancer cell lines are exposed to fever-range temperatures, they separate into thermo-tolerant and thermo-sensitive groups that do not consistently track tissue of origin, prompting the proposal that tumors might be classified by thermic predilection for patient stratification [16]. This proposed classification rests on a limited number of cell lines and awaits systematic validation. In colorectal carcinoma, cell lines differ in viability, cell death, and cell-cycle responses to heat, both alone and in combination with cytotoxic drugs [17,18]. Because the most developed cell-intrinsic evidence derives from colorectal cancer, we use colorectal cancer as the principal proof-of-concept model throughout this review, drawing on other tumor types where data allow and marking explicitly where generalization beyond colorectal cancer remains untested.

The response is not necessarily linear and may be biphasic, although direct, densely sampled evidence for a biphasic curve across the narrow 37–40 °C window is still limited and the concept should be treated as a working hypothesis. A step from 37 to 38 °C can, in an otherwise unstressed cell, modestly accelerate biochemical reactions and proliferation. Toward 39–40 °C, proteotoxic load rises, the heat-shock response engages through HSF1 and induction of HSP70 and HSP90 [19,20,21], and cells with less proteostatic reserve may cross from acceleration into arrest or death [22]. The crossover temperature may therefore provide a functional readout of proteostatic reserve [23]. Because faster-cycling lines differ in their distribution across cell-cycle phases [24], baseline proliferation rate is both a candidate correlate of sensitivity and a potential confounder that should be accounted for before genotype–phenotype associations are interpreted. Repeated or prolonged mild exposure can also induce thermotolerance, a transient, heat-shock-protein-dependent state of increased resistance that shifts the thermal response and complicates any single-temperature readout.

Genotype may help set this threshold. Heat-shock chaperones are co-opted by cancer cells to drive migration, an effect counterbalanced by heat-shock-inducible regulators of GSK-3β [25], indicating that the thermal response is integrated with oncogenic signaling rather than representing a purely generic stress response. Mutation-stratified models suggest that oncogenic context shapes both the magnitude and the direction of the response to heat and hyperthermic chemotherapy. In colorectal cancer cells carrying activating KRAS mutations together with constitutive WNT/β-catenin signaling, mild hyperthermia further raises ERK and WNT pathway activity, and because these pathways already operate near a growth-constraining threshold, the additional activation suppresses proliferation rather than promoting it; isogenic models in which mutant KRAS is knocked in or out confirm that the KRAS allele itself governs this heat sensitivity [26]. Oncogenic genotype also conditions the response to hyperthermic chemotherapy. Across colorectal lines differing in TP53, KRAS, and BRAF status, the cytotoxic effect of a given agent under hyperthermia varies by line, so that a drug with little activity in one mutational background can become effective in another when combined with heat [27]. The direction of the effect is therefore not fixed: the same temperature may restrain growth in one genotype while sensitizing another to chemotherapy, which is why intrinsic thermal behavior cannot be inferred from temperature alone without reference to the underlying driver mutations. Mismatch-repair status is another plausible modifier: microsatellite-unstable tumors carry a high mutational burden and may therefore face greater demands on protein homeostasis, while aneuploidy can impose an additional chronic proteotoxic burden [28,29]. These observations generate a testable prediction: intrinsic thermal sensitivity may constitute a stable, subtype-associated phenotype patterned by microsatellite instability, chromosomal instability, and common oncogenic drivers. Consensus molecular subtypes, by contrast, are defined transcriptionally rather than by mutation and belong to a separate, expression-based axis; transcriptomic features of the heat response, in particular chaperone and heat-shock-protein expression, are a plausible correlate of proteostatic reserve and thermal sensitivity that warrants systematic study [30]. To be explicit about the strength of evidence in this section: the engagement of HSF1-driven proteostasis, the KRAS- and WNT-dependent growth response, and the genotype-conditioned response to hyperthermic chemotherapy are experimentally established in colorectal models; the links to mismatch-repair status, chromosomal instability, and a stable subtype-associated thermal phenotype are reported associations or, in several cases, predictions proposed here that remain to be tested.

**Figure 2:**
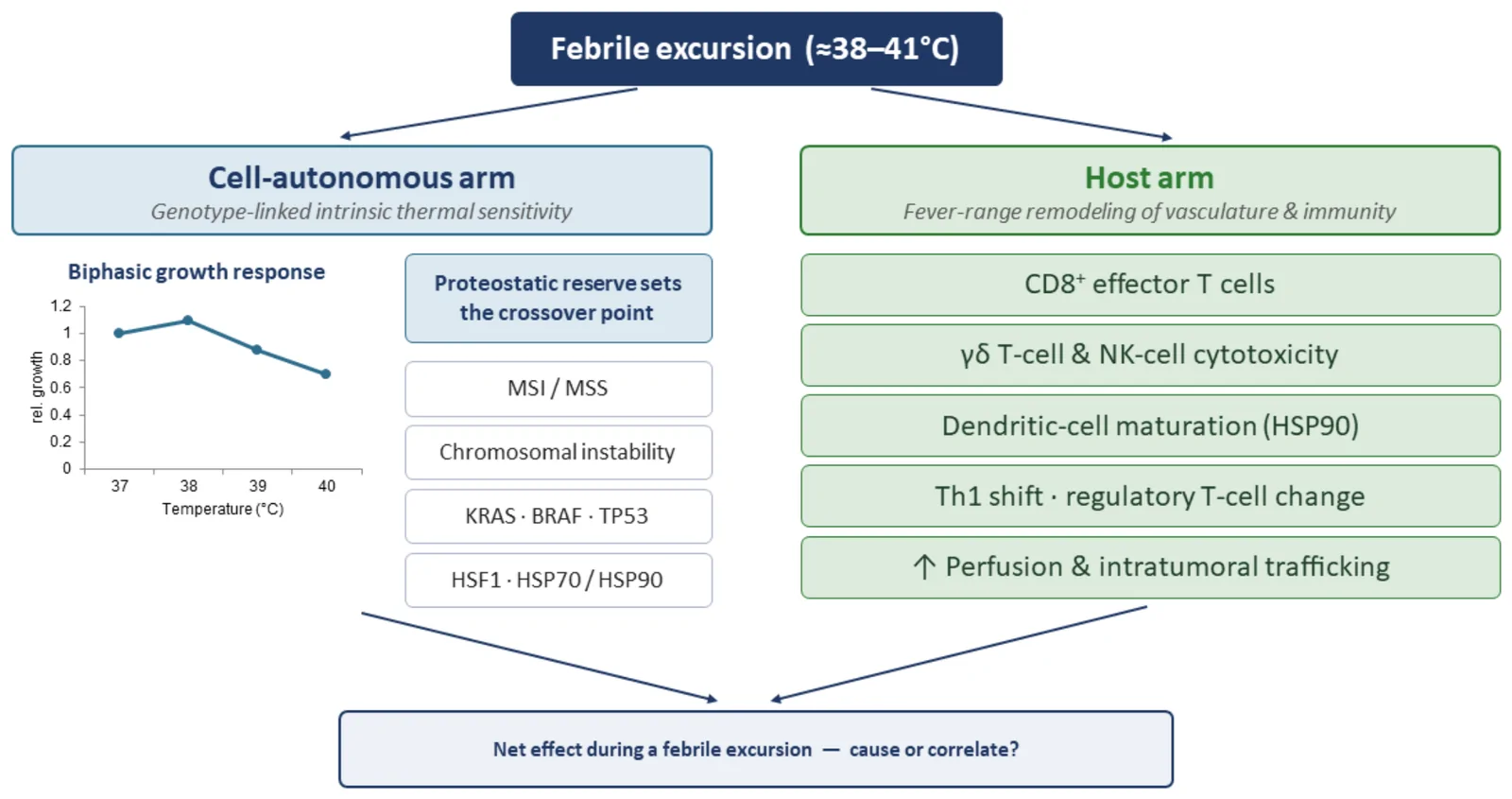
Two arms of the febrile excursion. Schematic contrasting the cell-autonomous arm (genotype-linked intrinsic thermal sensitivity, with a biphasic growth response across 37 to 40 °C set by proteostatic reserve) with the host arm (fever-range remodeling of vasculature and antitumor immunity), both converging on the net effect during a febrile excursion.

What is missing is a systematic map of this phenotype at true fever-range temperatures across a molecularly annotated panel, with the bidirectional growth response resolved into cytostasis versus cytotoxicity. This distinction matters because a tumor that regresses during fever and one that merely arrests imply different biology and different clinical stakes. The method of measurement is therefore critical: bulk biomass endpoints such as crystal violet cannot reliably distinguish a line that has stopped proliferating from one undergoing cell death, particularly because detached dead cells are lost during processing. Resolving these phenotypes requires live/dead or kinetic readouts and, ideally, patient-derived rather than extensively passaged models. This represents a clear open area in the field and one particularly amenable to patient-derived colorectal cancer models (Figure 2).

## 4. Fever-Range Temperatures Rewire Antitumor Immunity

Whatever mild warming does to an isolated tumor cell, in vivo that effect is embedded within a host response that is substantial at fever-range temperatures and increasingly well defined. Fever-range temperatures increase T-cell plasma-membrane fluidity and promote pre-clustering of receptor-signaling components, potentially lowering the activation threshold and favoring effector differentiation [31,32]. Recent work shows that febrile temperatures reprogram T-cell metabolism with subset-specific consequences, increasing mitochondrial mass and respiratory capacity in some compartments while inducing mitochondrial stress and DNA damage in others, suggesting that heat can both strengthen and prune the responding repertoire [5,33]. Among these populations, CD8^+^ effector T cells are of particular interest, since they are the principal mediators of antitumor cytotoxicity and their antigen-specific differentiation and effector function are enhanced by physiological-range heating [32,33]. Innate immunity is engaged as well: fever-range exposure promotes dendritic-cell maturation through induction of HSP90 [34]. Innate-like lymphocytes respond too; transient exposure to 40 °C potentiates the cytotoxicity of Vδ2^+^ γδ T cells through HSP70 upregulation [6]. Fever-range effects are therefore not uniformly beneficial; the same thermal stress that enhances some effector populations can impair others, making the net immunological consequence dependent on cell type, temperature, and exposure duration.

At the tissue level, mild heating increases perfusion and oxygenation and can promote immune-cell trafficking into tumors [31,35]. It can also increase tumor-cell surface expression of stress ligands such as MICA/B, which engage the activating NK-cell receptor NKG2D [36]. Heat further intersects with antigen-presentation pathways: heat-shock proteins exposed or released by stressed tumor cells can chaperone tumor antigens to dendritic cells and promote their maturation, while fever-range exposure can shift CD4+ T-cell responses toward a T helper 1 phenotype and alter regulatory T-cell function. These properties have driven interest in mild hyperthermia as an immunological adjuvant. Localized mild hyperthermia can promote immunogenic forms of tumor-cell stress and reshape the tumor microenvironment, with reported synergy with interleukin-2 or immune-checkpoint blockade in immunologically cold tumors [37]. Mild photothermal approaches combined with chemotherapy can likewise enhance dendritic-cell activation and antitumor immunity [38]. Systems-level modeling supports this dual action, predicting that transient hyperthermia can shift tumor–immune dynamics toward tumor control by simultaneously restraining tumor growth and enhancing immune recruitment [39]. Clinically, fever-range and systemic whole-body hyperthermia are being re-evaluated as adjuncts to chemotherapy, radiotherapy, and immunotherapy, with generally acceptable tolerability in early studies [7,8,9,40,41].

These effects are summarized in Table 1, which indicates for each immune or stromal compartment the temperature and exposure studied, the experimental setting, the principal effect, and whether the evidence derives from genuine endogenous fever or from externally imposed heating. The great majority of the mechanistic data come from imposed heating rather than from regulated fever, which bears directly on how far these findings can be extrapolated to naturally occurring febrile episodes.

Two cautions constrain any inference from these findings to naturally occurring fever. First, most of the evidence derives from externally induced heating, which does not reproduce the hypothalamic set-point shift or its accompanying neuroendocrine context. Second, and more consequentially, the host-mediated and cell-autonomous arms can point in the same direction and are therefore difficult to disentangle. A febrile excursion may suppress an immunogenic tumor both because its cells are intrinsically heat-sensitive and because fever mobilizes antitumor immunity. Distinguishing these mechanisms is not a nicety: it determines whether intrinsic thermal sensitivity is a cause of outcome or merely a correlate of it. In vitro, the two arms can be separated with relative ease; in clinical settings, the separation is considerably more difficult.

## 5. The Febrile Paradox: Protection Versus Prognosis

Carried into the clinic, the literature seems to contradict itself. On one side, fever is associated with protection. A history of febrile childhood infection has been linked to a reduced later risk of several cancers [42,43,44], with risk falling as infection frequency increases, and case-control analyses have reported fewer febrile infectious diseases in the histories of cancer patients than in matched controls [10,11]; these are retrospective associations that concern infection history rather than any measured temperature. Such associations need not reflect the temperature of past fevers directly; they may instead indicate trained innate immunity or a state of heightened immune surveillance that detects transformed cells earlier [45]. This tradition extends the oldest observation in the field: the coincidence of spontaneous tumor regression with acute febrile infection that inspired Coley [46,47,48]. Modern experimental models have revisited this phenomenon: intratumoral injection of live *Streptococcus pyogenes* induced regression of pancreatic tumors in a syngeneic model [49], bacterial and avitalized-bacterial approaches can control tumor growth through activation of innate immunity [50,51], and spontaneous regression continues to be associated with microbial infection [52]. These bacterial and Coley-type regressions are driven chiefly by the immune response to the pathogen and secondarily to the tumor; a specific causal contribution of the accompanying fever or temperature elevation has not been established, and fever and antipyretic use were frequently undocumented in the historical reports. On the other side, among patients already diagnosed with cancer and undergoing surgery, postoperative fever is associated with poorer survival and higher recurrence rates [53,54]. Taken at face value, the two bodies of evidence cannot both mean that fever benefits patients with cancer.

**Figure 3:**
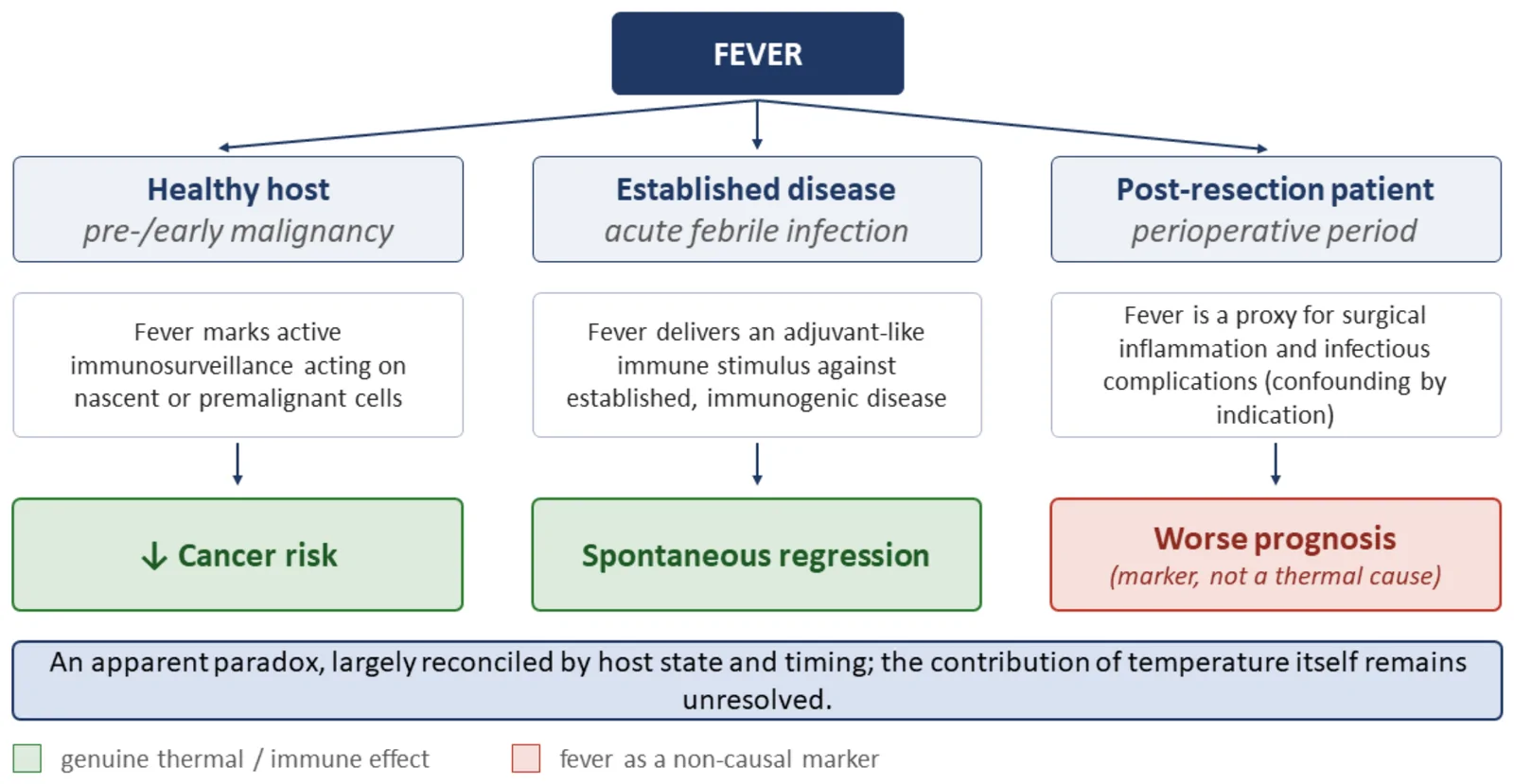
Resolving the febrile paradox. The same term “fever” indexes different biology across host state and timing: immunosurveillance in a healthy host (lower risk), an adjuvant-like stimulus against established immunogenic disease (regression), and a proxy for surgical inflammation after resection (worse prognosis). The reconciliation is presented as a hypothesis-generating framework rather than a resolved one.

The apparent contradiction is substantially reconciled, though not fully dissolved, once fever is disaggregated (Figure 3). The word is doing three incompatible jobs. In the risk literature, fever marks an immune response occurring before or during the emergence of malignancy and may therefore serve as an index of immune surveillance. In the regression tradition, fever accompanies an acute infection that provides a powerful adjuvant-like stimulus in the setting of established, potentially immunogenic disease. In the prognosis literature, postoperative fever is not an isolated thermal exposure but a marker of surgical inflammation, tissue injury, or infectious complications, a setting in which the fever may reflect the severity of the underlying process rather than directly cause the poorer outcome. The discriminating variables are the state of the host, the timing relative to malignancy, and what the fever represents. Treating fever as a single exposure can therefore create a paradox that is substantially attenuated when these contexts are separated, while obscuring the biological question they share. We therefore present the febrile paradox as a hypothesis-generating framework rather than a resolved one: the apparent contradiction can be reconciled by considering host state and timing, but whether temperature elevation itself contributes materially to any of these outcomes, as opposed to the infection, inflammation, or immune activation that accompanies it, remains empirically unresolved.

This reframing also exposes how limited the modern prospective evidence remains. The protective associations rest largely on retrospective and potentially recall-prone designs that predate modern prospective ascertainment. The prognostic signal, by contrast, is intrinsically vulnerable to confounding because postoperative fever is closely linked to the complications and inflammatory burden of recovery. One modifier runs through both sides and is rarely captured in sufficient detail: antipyretic use. If a febrile excursion is biologically active, routinely suppressing it could theoretically blunt an immunosurveillance benefit or, conversely, attenuate a harmful inflammatory response. Neither literature yet isolates the effect of a genuine febrile excursion on an established tumor. We emphasize that these considerations are scientific, not clinical: the evidence reviewed here does not justify any change to the management of fever in patients with cancer, where fever is appropriately treated for patient comfort and safety, and the hypothesis that fever suppression might influence antitumor immunity remains untested in that setting.

## 6. Cause or Correlate?

The two arms now converge on one question. Intrinsic thermal sensitivity is real, heterogeneous, and potentially genotype-linked. Fever-range remodeling of the microenvironment is also real and can be immunostimulatory, although its effects are context-dependent. Both plausibly shape how a tumor fares during a febrile excursion. The open issue is their relative weight: is the tumor cell’s intrinsic thermal sensitivity a cause of outcome during fever, or a correlate carried along by, and confounded with, the host immune response?

Several considerations suggest that the cell-autonomous effect, so readily detected in vitro, may be the weaker force in vivo. At fever-range temperatures, direct thermal cytotoxicity is generally limited, whereas the host mounts a coordinated vascular, neuroendocrine, and immune response. A cell that divides slightly faster at 38 °C in the absence of an immune system need not gain a corresponding advantage in a patient. In vivo, the febrile excursion superimposes a modest cell-autonomous effect on a potentially dominant host response. The confounding may be particularly strong in molecularly defined settings. A microsatellite-unstable, highly immunogenic tumor might be suppressed during fever both because its cells are intrinsically more heat-sensitive and because fever amplifies immunity against an antigen-rich target. An in vivo correlation between thermal phenotype and outcome cannot, by itself, distinguish these mechanisms; doing so requires designs that hold relevant tumor features constant or measure thermal sensitivity and immune context independently.

The experimental implications follow directly. Mismatch-repair-corrected isogenic pairs could help separate cell-autonomous proteostatic effects from effects associated with immunogenicity. Immunocompetent and humanized models could test whether an in vitro thermal phenotype persists in the presence of an intact or human immune compartment. Finally, correlating thermal phenotype with febrile history and clinical outcome, stratified by molecular subtype and immune context, could help distinguish a cause from a correlate.

Framing the question in this way turns a vague intuition, that tumors with different thermal responses may behave differently during fever, into a falsifiable hypothesis and defines the experiments needed to test it.

## 7. Toward Clinical Translation and Experimental Standardization

If intrinsic thermal sensitivity can be measured reliably, its potential clinical relevance is multi-factorial and should not be reduced to a single scenario. At least three distinct questions arise. First, in the setting of therapeutic hyperthermia, a tumor’s thermal phenotype could inform whether local or whole-body heating is likely to help or to be futile, and at what thermal dose. Second, for naturally occurring fever, the direction of a tumor’s intrinsic heat response (growth-promoting versus growth-restraining) may be a variable worth investigating, since positive and negative heat responders might behave differently during febrile episodes. We raise this only to flag tumor thermal phenotype as a candidate variable for future study in the context of naturally occurring fever; the present evidence does not support any speculation about altering fever management, and we make none. Third, thermal phenotype may matter chiefly in combination with treatment, modifying the response to chemotherapy or radiotherapy delivered during a thermal excursion. These scenarios involve different biology and rest on different levels of evidence, and we present them as hypotheses to be tested rather than as clinical guidance. Whether thermal phenotyping would add information beyond established molecular markers such as KRAS mutation and mismatch-repair status is itself one of the questions to be resolved.

Realizing any of this requires that fever-range thermal biology be studied under reported, controlled conditions. If temperature is to become an explicit experimental variable, it should be treated with the same rigor as a drug concentration. At minimum, studies should report the actual sample temperature rather than the nominal incubator setting; the exposure duration together with the heating and cooling kinetics; whether exposure was single, repeated, or continuous; and the recovery interval before readout, since repeated or prolonged mild exposure induces thermotolerance that shifts the response. Protocols should be compared by thermal dose rather than by peak temperature alone [15]. Endpoints should distinguish cytostatic from cytotoxic effects using live/dead or kinetic readouts rather than bulk-biomass assays alone, and matched normothermic and handling controls should accompany every heated condition. Adopting these minimal standards would make results comparable across laboratories and convert the proposed experimental agenda into a tractable, reproducible program.

## 8. Conclusions and Future Directions

Temperature deserves to be treated as a variable in cancer biology, not as a constant to be assumed. The mild fever-range excursion, distinct from both externally induced hyperthermia and any assumed steady thermal niche, links an intrinsic, potentially genotype-associated property of tumor cells to a well-developed fever immunobiology and to a clinical record whose apparent contradictions are substantially reconciled, though not fully resolved, once fever is disaggregated by host state and timing. The immediate priorities are concrete: map intrinsic thermal sensitivity at fever-range temperatures across molecularly annotated tumor panels and determine whether it predicts outcome beyond established subtype markers; resolve the biphasic growth response into cytostasis and cytotoxicity and define its proteostatic basis; separate the cell-autonomous and host-mediated arms in vivo; replace recall-based fever epidemiology with prospective ascertainment that accounts for host state and timing; and test the difference between naturally occurring fever and externally induced heating as a biological variable in its own right. None of this requires exotic technology. Much of it requires chiefly that temperature be measured and controlled rather than ignored, although disentangling the cell-autonomous and systemic-fever contributions in vivo is experimentally more demanding and will depend on the isogenic and immunocompetent designs outlined above. Colorectal cancer, with its well-defined molecular subtypes and mature panels of patient-derived models, is an unusually tractable setting in which to begin, and we have used it as the principal proof-of-concept model here. Whether intrinsic thermal sensitivity proves to be a cause or a correlate, resolving this question could convert a century of scattered observations into a coherent thermal dimension of cancer biology.

## Figures and Tables

**Table 1:** Representative immune and stromal effects of fever-range temperatures, with experimental setting and evidence type. Most entries derive from externally imposed heating rather than from regulated endogenous fever.

Compartment/Cell Type	Temperature & Exposure	Setting	Principal Effect	Evidence Type	Ref.
CD8^+^ effector T cells	~38.5–39.5 °C, hours to days	In vitro (human, mouse)	Enhanced effector differentiation and function; increased mitochondrial translation	Imposed fever-range heating	[32,33]
T-cell subsets (metabolism)	Febrile range	In vitro and in vivo (fever, inflammation)	Subset-specific mitochondrial gain versus stress and DNA damage	Endogenous fever and imposed	[5]
Vδ2^+^ γδ T cells	Transient 40 °C	In vitro (human)	Increased cytotoxicity via HSP70 upregulation	Imposed	[6]
Dendritic cells	Fever-like (~39–40 °C)	In vitro	Maturation via induction of HSP90	Imposed	[34]
NK cells	Fever-range (~39.5 °C)	In vitro	Increased NKG2D-dependent cytotoxicity; MICA/B on targets	Imposed	[36]
Tumor microvasculature; T-cell trafficking	Fever-range whole-body heating	In vivo (mouse, human)	IL-6 trans-signaling opens trafficking gateways; increased perfusion	Imposed (whole-body)	[31,35]
Tumor microenvironment (localized)	Localized mild hyperthermia (gold nanorods)	In vivo (mouse, cold tumors)	Immunogenic cell death; synergy with IL-2 or checkpoint blockade	Imposed (localized)	[37]
Tumor microenvironment (photothermal)	Mild photothermal plus chemotherapy	In vivo (mouse)	Enhanced dendritic-cell activation and antitumor immunity	Imposed (photothermal)	[38]
Tumor–immune dynamics	Transient mild hyperthermia	In silico	Predicted shift toward tumor control	Computational model	[39]

## Data Availability

No new data were created or analyzed in this study. Data sharing is not applicable to this article.

## References

[1] Pokharel A., Luitel N., Khatri A., Khadka S., Shrestha R. (2024). Review on the evolving role of infrared thermography in oncological applications. Infrared Phys. Technol..

[2] Chrétien D., Bénit P., Ha H.H., Keipert S., El-Khoury R., Chang Y.-T., Jastroch M., Jacobs H.T., Rustin P., Rak M. (2018). Mitochondria are physiologically maintained at close to 50 °C. PLoS Biol..

[3] Lane N. (2018). Hot mitochondria?. PLoS Biol..

[4] Baffou G., Rigneault H., Marguet D., Jullien L. (2014). A critique of methods for temperature imaging in single cells. Nat. Methods.

[5] Heintzman D.R., Sinard R.C., Fisher E.L., Ye X., Patterson A.R., Elasy J.H., Voss K., Chi C., Sugiura A., Rodriguez-Garcia G.J. (2024). Subset-specific mitochondrial stress and DNA damage shape T cell responses to fever and inflammation. Sci. Immunol..

[6] Lin L., Chen Y., Chen D., Shu J., Hu Y., Yin Z., Wu Y. (2022). Transient 40 °C-shock potentiates cytotoxic responses of Vδ2+ γδ T cells via HSP70 upregulation. Cancer Immunol. Immunother..

[7] Brancato L., Gorbaslieva I., Rudenko O., Logghe T., van Zwol E., Boulet G., Van den Bossche L., Van den Bossche J., Peeters M., Chapelle T. (2026). The MATTERS trial: Safety and tolerability of whole-body hyperthermia at 41.50 °C in combination with chemotherapy in metastatic cancer patients. Cancer Res. Commun..

[8] Faghihi Moghaddam F., Bakhshandeh M., Mofid B., Sahinbas H., Faeghi F., Mirzaei H., Rakhsha A., Yousefi Kashi A.S., Sadeghi R., Mahdavi A. (2024). Clinical effectiveness of combined whole body hyperthermia and external beam radiation therapy versus EBRT alone in patients with painful bony metastases: A phase III clinical trial. J. Therm. Biol..

[9] Liu P., Wu J., Chen L., Wu Z., Wu Y., Zhang G., Yu B., Zhang B., Wei N., Shi J. (2024). Water-filtered infrared A radiation hyperthermia combined with immunotherapy for advanced gastrointestinal tumours. Cancer Med..

[10] Albonico H.U., Bräker H.U., Hüsler J. (1998). Febrile infectious childhood diseases in the history of cancer patients and matched controls. Med. Hypotheses.

[11] Kleef R., Jonas W.B., Knogler W., Stenzinger W. (2001). Fever, cancer incidence and spontaneous remissions. Neuroimmunomodulation.

[12] Kluger M.J. (1992). Fever revisited. Pediatrics.

[13] Gordon C.J. (1983). A review of terms for regulated vs. forced, neurochemical-induced changes in body temperature. Life Sci..

[14] Mackowiak P.A., Boulant J.A. (1996). Fever’s glass ceiling. Clin. Infect. Dis..

[15] Sapareto S.A., Dewey W.C. (1984). Thermal dose determination in cancer therapy. Int. J. Radiat. Oncol. Biol. Phys..

[16] Kalamida D., Karagounis I.V., Mitrakas A., Kalamida S., Giatromanolaki A., Koukourakis M.I. (2015). Fever-range hyperthermia vs. hypothermia effect on cancer cell viability, proliferation and HSP90 expression. PLoS ONE.

[17] Petrakis G., Mantso T., Koukourakis M.I., Panayiotidis M.I., Botaitis S. (2022). Effectiveness of hyperthermia as monotherapy and adjuvant therapy approaches against an in vitro model of colorectal carcinoma. Anticancer Res..

[18] Helderman R.F.C.P.A., Löke D.R., Verhoeff J., Rodermond H.M., van Bochove G.G.W., Boon M., van Kesteren S., Garcia Vallejo J.J., Kok H.P., Tanis P.J. (2020). The temperature-dependent effectiveness of platinum-based drugs mitomycin-C and 5-FU during hyperthermic intraperitoneal chemotherapy (HIPEC) in colorectal cancer cell lines. Cells.

[19] Home T., Jensen R.A., Rao R. (2015). Heat shock factor 1 in protein homeostasis and oncogenic signal integration. Cancer Res..

[20] Dai C., Sampson S.B. (2016). HSF1: Guardian of proteostasis in cancer. Trends Cell Biol..

[21] Wang G., Cao P., Fan Y., Tan K. (2020). Emerging roles of HSF1 in cancer: Cellular and molecular episodes. Biochim. Biophys. Acta Rev. Cancer.

[22] de Andrade Mello P., Bian S., Savio L.E.B., Zhang H., Zhang J., Junger W., Wink M.R., Lenz G., Buffon A., Wu Y. (2017). Hyperthermia and associated changes in membrane fluidity potentiate P2X7 activation to promote tumor cell death. Oncotarget.

[23] Ahmed K., Zaidi S.F., Mati-ur-Rehman, Rehman R., Kondo T. (2020). Hyperthermia and protein homeostasis: Cytoprotection and cell death. J. Therm. Biol..

[24] Bhuyan B.K., Day K.J., Edgerton C.E., Ogunbase O. (1977). Sensitivity of different cell lines and of different phases in the cell cycle to hyperthermia. Cancer Res..

[25] Koizumi K., Domoto T., Minamoto T., Satomura K., Nakajima H. (2023). Deactivation of glycogen synthase kinase-3β by heat shock-inducible tumor small protein attenuates hyperthermia-induced pro-migratory activity in colorectal cancer cells. Int. J. Oncol..

[26] Bordonaro M., Shirasawa S., Lazarova D.L. (2016). In hyperthermia increased ERK and WNT signaling suppress colorectal cancer cell growth. Cancers.

[27] Kabagwira J., Fuller R.N., Vallejos P.A., Sugiono C.S., Andrianarijaona V.-M., Chism J.B., O’Leary M.P., Caba Molina D., Langridge W., Senthil M. (2024). Amplifying curcumin’s antitumor potential: A heat-driven approach for colorectal cancer treatment. Onco Targets Ther..

[28] Donnelly N., Storchová Z. (2015). Aneuploidy and proteotoxic stress in cancer. Mol. Cell. Oncol..

[29] Amponsah P.S., Storchová Z. (2025). The proteostasis burden of aneuploidy. Biol. Chem..

[30] Scutigliani E.M., Lobo-Cerna F., Mingo Barba S., Scheidegger S., Krawczyk P.M. (2023). The effects of heat stress on the transcriptome of human cancer cells: A meta-analysis. Cancers.

[31] Evans S.S., Repasky E.A., Fisher D.T. (2015). Fever and the thermal regulation of immunity: The immune system feels the heat. Nat. Rev. Immunol..

[32] Mace T.A., Zhong L., Kilpatrick C., Zynda E., Lee C.T., Capitano M., Minderman H., Repasky E.A. (2011). Differentiation of CD8+ T cells into effector cells is enhanced by physiological range hyperthermia. J. Leukoc. Biol..

[33] O’Sullivan D., Stanczak M.A., Villa M., Uhl F.M., Corrado M., Klein Geltink R.I., Sanin D.E., Apostolova P., Rana N., Edwards-Hicks J. (2021). Fever supports CD8+ effector T cell responses by promoting mitochondrial translation. Proc. Natl. Acad. Sci. USA.

[34] Basu S., Srivastava P.K. (2003). Fever-like temperature induces maturation of dendritic cells through induction of hsp90. Int. Immunol..

[35] Fisher D.T., Chen Q., Skitzki J.J., Muhitch J.B., Zhou L., Appenheimer M.M., Vardam T.D., Weis E.L., Passanese J., Wang W.-C. (2011). IL-6 trans-signaling licenses mouse and human tumor microvascular gateways for trafficking of cytotoxic T cells. J. Clin. Investig..

[36] Ostberg J.R., Dayanc B.E., Yuan M., Oflazoglu E., Repasky E.A. (2007). Enhancement of natural killer (NK) cell cytotoxicity by fever-range thermal stress is dependent on NKG2D function and is associated with plasma membrane NKG2D clustering and increased expression of MICA on target cells. J. Leukoc. Biol..

[37] Kennedy B.E., Noftall E.B., Dean C., Roth A., Clark K.N., Rowles D., Singh K., Pagliaro L., Giacomantonio C.A. (2025). Targeted intra-tumoral hyperthermia using uniquely biocompatible gold nanorods induces strong immunogenic cell death in two immunogenically ‘cold’ tumor models. Front. Immunol..

[38] Yi Q., He S., Liao K., Yue Z., Mei L. (2024). Nanoparticles integrated with mild photothermal therapy and oxaliplatin for tumor chemotherapy and immunotherapy. Nanomedicine.

[39] Barrera-León A.S., Rodríguez-Quiñones L., De Mendoza A.M. (2025). Impact of mild hyperthermia on tumor-immune dynamics explored through mathematical modeling. Sci. Rep..

[40] Hohneck A.L., Sadikaj L., Heinemann L., Schroeder M., Riess H., Gerhards A., Burkholder I., Heckel-Reusser S., Gottfried J., Hofheinz R.D. (2023). Patients with advanced pancreatic cancer treated with mistletoe and hyperthermia in addition to palliative chemotherapy: A retrospective single-center analysis. Cancers.

[41] Sulyok I., Fleischmann E., Stift A., Roth G., Lebherz-Eichinger D., Kasper D., Spittler A., Kimberger O. (2012). Effect of preoperative fever-range whole-body hyperthermia on immunological markers in patients undergoing colorectal cancer surgery. Br. J. Anaesth..

[42] Kölmel K.F., Gefeller O., Haferkamp B. (1992). Febrile infections and malignant melanoma: Results of a case-control study. Melanoma Res..

[43] Kölmel K.F., Pfahlberg A., Mastrangelo G., Niin M., Botev I.N., Seebacher C., Schneider D., Lambert D., Shafir R., Kokoschka E.M. (1999). Infections and melanoma risk: Results of a multicentre EORTC case-control study. Melanoma Res..

[44] Hoption Cann S.A., van Netten J.P., van Netten C. (2006). Acute infections as a means of cancer prevention: Opposing effects to chronic infections?. Cancer Detect. Prev..

[45] Netea M.G., Domínguez-Andrés J., Barreiro L.B., Chavakis T., Divangahi M., Fuchs E., Joosten L.A.B., van der Meer J.W.M., Mhlanga M.M., Mulder W.J.M. (2020). Defining trained immunity and its role in health and disease. Nat. Rev. Immunol..

[46] Wiemann B., Starnes C.O. (1994). Coley’s toxins, tumor necrosis factor and cancer research: A historical perspective. Pharmacol. Ther..

[47] Hoption Cann S.A., van Netten J.P., van Netten C. (2003). Dr William Coley and tumour regression: A place in history or in the future. Postgrad. Med. J..

[48] Hobohm U. (2005). Fever therapy revisited. Br. J. Cancer.

[49] Maletzki C., Linnebacher M., Kreikemeyer B., Emmrich J. (2008). Pancreatic cancer regression by intratumoural injection of live Streptococcus pyogenes in a syngeneic mouse model. Gut.

[50] Linnebacher M., Maletzki C., Klier U., Klar E. (2012). Bacterial immunotherapy of gastrointestinal tumors. Langenbecks Arch. Surg..

[51] Klier U., Maletzki C., Göttmann N., Kreikemeyer B., Linnebacher M. (2011). Avitalized bacteria mediate tumor growth control via activation of innate immunity. Cell. Immunol..

[52] Kucerova P., Cervinkova M. (2016). Spontaneous regression of tumour and the role of microbial infection—Possibilities for cancer treatment. Anticancer Drugs.

[53] Feng F., Tian Y., Yang X., Sun L., Hong L., Yang J., Guo M., Lian X., Fan D., Zhang H. (2017). Postoperative fever predicts poor prognosis of gastric cancer. Oncotarget.

[54] Yan T., Yin W., Zhou L., Jiang Y., Shen Z., Shao Z., Lu J. (2010). Postoperative fever: The potential relationship with prognosis in node negative breast cancer patients. PLoS ONE.

